# Avian influenza and gut microbiome in poultry and humans: A “One Health” perspective

**DOI:** 10.1016/j.fmre.2023.10.016

**Published:** 2023-12-27

**Authors:** Ling Zhang, Erkai Zhou, Ce Liu, Xiaoyu Tian, Baode Xue, Kai Zhang, Bin Luo

**Affiliations:** a Institute of Occupational Health and Environmental Health, School of Public Health, Lanzhou University, Lanzhou, Gansu 730000, China; b Department of Environmental Health Sciences, School of Public Health, University at Albany, State University of New York, Rensselaer, NY 12144, USA; c Shanghai Key Laboratory of Meteorology and Health, Shanghai Meteorological Bureau, Shanghai 200030, China; d Shanghai Typhoon Institute, China Meteorological Administration, Shanghai 200030, China

**Keywords:** Avian influenza, AIV, Poultry, Gut microbiome, One Health, Poultry-Environment-Human Interface

## Abstract

A gradual increase in avian influenza outbreaks has been found in recent years. It is highly possible to trigger the next human pandemic due to the characteristics of antigenic drift and antigenic shift in avian influenza virus (AIV). Although great improvements in understanding influenza viruses and the associated diseases have been unraveled, our knowledge of how these viruses impact the gut microbiome of both poultry and humans, as well as the underlying mechanisms, is still improving. The “One Health” approach shows better vitality in monitoring and mitigating the risk of avian influenza, which requires a multi-sectoral effort and highlights the interconnection of human health with environmental sustainability and animal health. Therefore, monitoring the gut microbiome may serve as a sentinel for protecting the common health of the environment, animals, and humans. This review summarizes the interactions between AIV infection and the gut microbiome of poultry and humans and their potential mechanisms. With the presented suggestions, we hope to address the current major challenges in the surveillance and prevention of microbiome-related avian influenza with the “One Health” approach.

## Highlights

• Avian influenza is a zoonotic disease and should be considered under the “One Health” framework to prevent and response transmission from animal hosts to humans.
• There is an interaction between the poultry or human gut microbiome and AIV infection.
• The implementation of a surveillance program for the poultry or human gut microbiome might serve as a sentinel for monitoring the overall risk of AIV infection.

## 1 Introduction

Since the emergence of influenza virus, it has caused four devastating pandemics globally in the last 100 years or more [1]. The worst of these was the 1918 H1N1 pandemic, which was estimated to have caused at least 50 million deaths worldwide [2]. H5, H7, and H9 subtypes are the most common and risky types of avian influenza virus (AIV) that have caused frequent human outbreaks due to their characteristics of antigenic drift and antigenic shift, which may also have the potentiality to trigger a large-scale human pandemic in the future [3]. The majority of fatal human avian influenza infections have been reported to be tied with the H7 and H5 subtypes of the AIV. It often exhibits severe symptoms (such as pneumonia, multiorgan dysfunction, and cytokine dysregulation) [4] and has high case fatality rates (up to 40%–60%) [5,6]. These facts indicate that avian influenza may pose a serious threat to global public health, so exploring strategies for preventing and dealing with avian influenza outbreaks is still worth exploring.

Antibiotics are commonly used in agriculture to prevent and treat pathogenic infections in poultry and to mitigate large-scale outbreaks of infectious diseases [7]. However, due to the ever-higher rates of antibiotic use and selective pressure for resistance, the efficacy of these drugs is declining worldwide [8,9]. Although vaccines play an important role in avian influenza control and prevention of the related infection, they are not “magic bullets” for eliminating AIV or eradicating the disease [10]. In addition, previous work has found that the implementation of control measures combining culling and vaccines has failed to rapidly eradicate highly pathogenic avian influenza outbreaks [11]. These approaches are less likely to be the best way to treat and prevent AIV infection, which neglects the overall Poultry-Environment-Human dimension [12]. Besides, we are gradually realizing that microbiomes are crucial in “One Health” because they connect each of these components [13]. Microbiomes can directly or indirectly influence human, animal, and environmental health, thereby promoting “One Health” [14].

Balancing the gut microbiome has been considered essential for creating colonization resistance to pathogens (viruses, bacteria, parasites) [15]. Previous studies have shown that AIV could affect the composition and integrity of the host gut microbiome during infection [16,17]. Furthermore, the microbiome modulates the host immune response to AIV infections [18], suggesting a reciprocal regulation between AIV infection and the microbiome. Therefore, by manipulating the gut microbiome of poultry and human hosts, it is likely to enhance their resistance and resilience to AIV infections. Although more and more attentions have been recently paid to the interactions of Host-Microbiome-AIV as a critical link to the control of AIV infectivity and pathogenicity, the effects of gut microbiome regulation on AIV-infected hosts are still worth extensively exploring. Meanwhile, most studies have investigated the relationship between host microbiome and AIV infection from a one-way viewpoint, although results from these studies have contributed significantly to the understanding of the dynamic and complex relationship between the gut microbiome and AIV infection. While, most studies have been confined to mouse models [19,20]. Some key differences among the different systems still need to be taken into account (such as the specific composition of the human and avian gut microbiomes, and the environmental factors that humans and poultry are exposed to are quite distinct from mouse) [19,21], and translating these findings from mouse models to humans and avian remains nontrivial.

In recent years, the spread and outbreaks of avian influenza have increased [22] due to global trade, poultry production, climate change, bird migration, and human movement. Therefore, effectively reducing the transmission and incidence of avian influenza is an interregional, intersectoral, and interdisciplinary endeavor. Experts have long called for a worldwide “One Health” approach to avian influenza. The “One Health” approach is based on the premise that human health is linked to environmental sustainability and animal health. It has the potential to establish a novel framework that engages professionals from diverse fields, including veterinarians, physicians, public health scientists, ecologists, and economists [23]. Such a multidisciplinary approach could enhance cooperation across countries and regions in combating the epidemic of avian influenza [23].

**Fig. 1:**
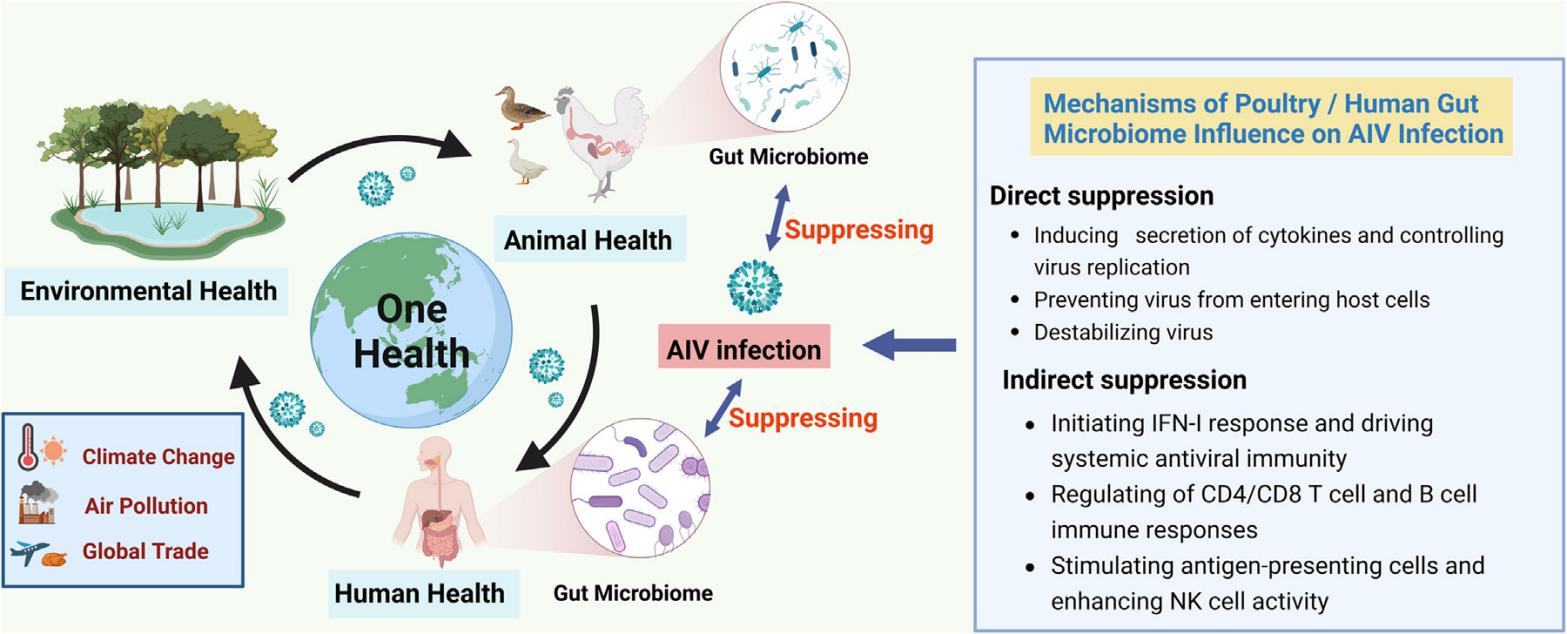
**Mechanism of gut microbiome on AIV infection through the Poultry-Environment-Human Interface based on “One Health” approach.** Note: The “One Health” approach links human health, animal health, and environmental health, which highlights the integration and overall health and is affected by climate change, air pollution, global trade, and so on. There are interactions between AIV infection and the intestinal microbiome in poultry and/or humans. Poultry and/or human gut microbiome can directly or indirectly suppress AIV infection, and when AIV infects poultry and or humans, changes in the composition and function of their gut microbiome are found.

In this review, we discussed the associations and mechanisms linking the gut microbiome of poultry and humans to AIV infection from a “One Health” perspective (Fig. 1). We also provided suggestions based on the “One Health” approach to address the current major challenges in surveillance and prevention of avian influenza.

## 2 Avian influenza in the Poultry-Environment-Human Interface and the role of the gut microbiome

The links between humans, animals, and the environment are becoming stronger, particularly the current poultry and human influenza outbreaks, so the challenge of controlling avian influenza is more complex than ever. Implementing a gut microbiome surveillance program may act as a sentinel for monitoring AIV infection.

### 2.1 Poultry-environment interface

There is growing evidence that the gut microbiome plays an important role in disease control and immunomodulation [15,24]. The gut microbiome of poultry is complex and dynamic, and gut function is influenced by the composition and diversity of bacteria [25]. Based on the 16S rRNA gene targeting sequence analysis, the chicken gut microbiome was found to be dominated by Firmicutes, Bacteroidetes, Proteobacteria, and Actinobacteria in 13 bacterial phyla, accounting for > 90% of the sequences. The main representative phylum of the healthy ducks and geese gut microbiome were Firmicutes, Proteobacteria, Bacteroidetes, Cyanobacteria, and Actinobacteria [26,27], and Bacteroidetes were more abundant in the cecum than in other parts [28].

AIV infection significantly alters the composition of the gut microbiome [29,30], and can even disrupt the gut function [29,32], leading to immune dysregulation (Table 1). In H9N2 AIV-infected chickens, AIV promotes the depletion of probiotic genera including *Lactobacillus, Enterococcus*, and *Streptococcus*, and the enrichment of *Proteobacteria*-associated pathogenic bacteria (such as *Escherichia coli, Salmonella*) [29,30]. In addition, studies have found that inflammatory responses and intestinal damage occur in AIV-infected poultry [29,^32^,34]. When chickens are infected with the AIV, the mRNA expression of tight junction proteins (ZO-1, claudin 3, and occludin), trefoil factor 2 (TFF2), and recombinant mucin 2 (Muc2) could significantly reduce, damaging the integrity of the intestine [29]. Additionally, accompanied by a significant upregulation of mRNA expression of pro-inflammatory cytokines, such as Interferon-gamma (IFN-γ), Interleukin 22 (IL-22), IFN-α, and IL-17A in intestinal epithelial cells, there are also intestinal inflammations [29,32]. At the same time, bacteria are usually the secondary invaders during influenza infection, and overlapping bacterial infections can lead to accelerated disruption of the physical barrier of the gut, dysregulation of the innate immune response, and delayed restoration of microbiota homeostasis [34]. Therefore, AIV infection impairs the stability of the gut microbiological environment of poultry and even threatens health.

**Table 1:** **Effect of AIV infection on the gut microbiome of poultry**.

	Effect of AIV infection on the gut microbiome	References
Composition of the gut microbiome	Decreasing probiotics	[29,30]
	Increasing number of pathogenic bacteria	[29,31]
Function of the gut microbiome	Occurring inflammatory reaction	[32]
	Developing gut damage	[33]
	Increasing secondary infections	[34]

**Fig. 2:**
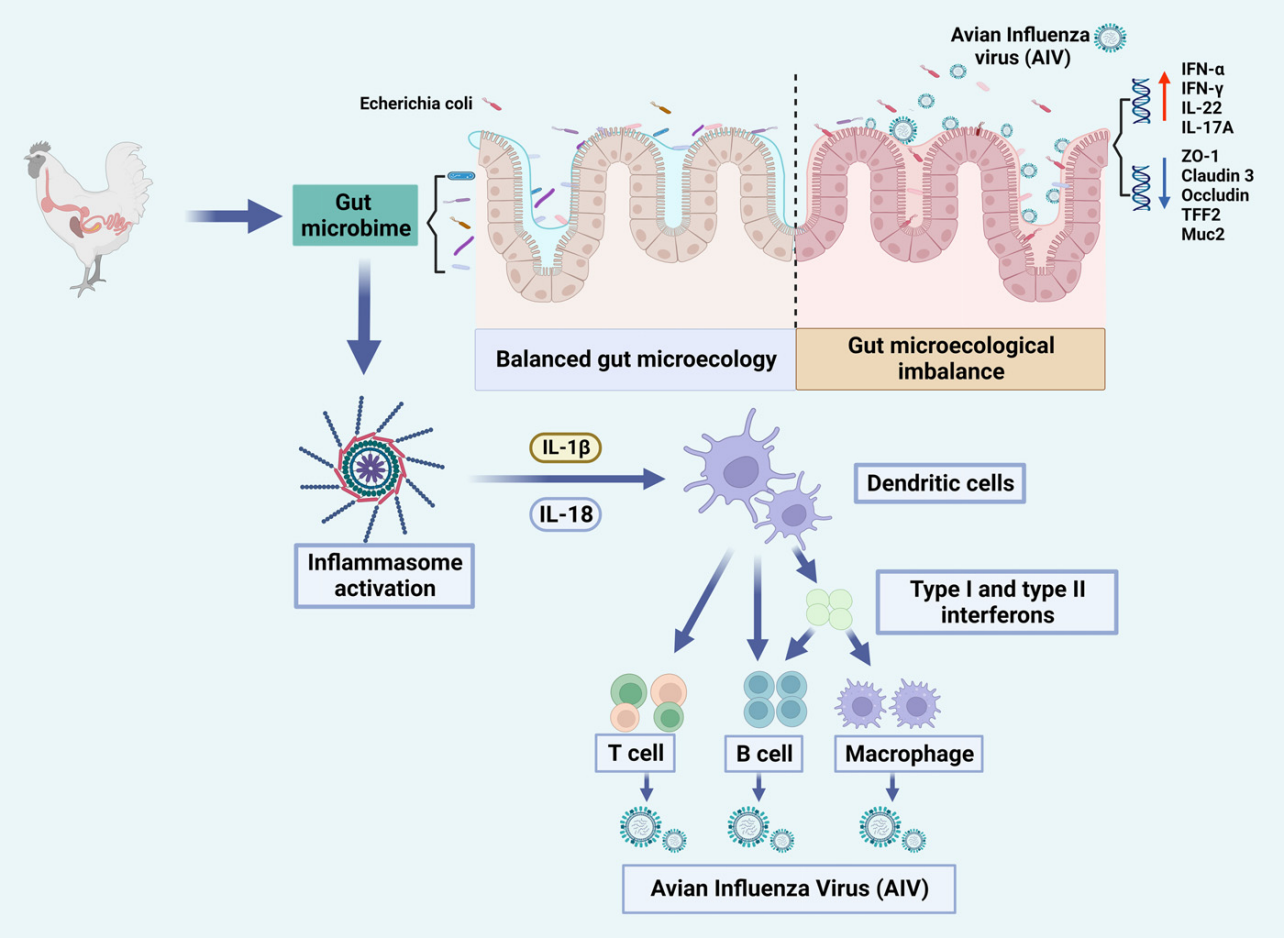
**The interactions between AIV infection and the gut microbiome of poultry in both healthy and pathological situations, using chickens as an example.** Note: IFN-γ represents Interferon gamma; IL-22 represents Interleukin-22; IL-17A represents Interleukin-17A; IL-1βrepresents Interleukin-1β; IL-18 represents Interleukin-18; ZO-1 represents Zonaoccludens 1; TFF2 represents Trefoil factor 2; Muc2 represents Recombinant Mucin 2.

Furthermore, the gut microbiome could modulate the poultry's immune response to AIV, either directly or indirectly, thereby suppressing infection (Table 2). On one hand, a balanced gut microbiome in poultry can help suppress AIV replication and reduce viral load [35,36]. The interaction between *Lactobacillus* and macrophages in the gut not only induces the secretion of various cytokines (including IL-1β, IFN-γ, and IFN-α cytokines) [42] but also enhances the antiviral response to AIV [43]. It has been proposed that *Enterococcus faecium* can prevent the virus from entering the cell by absorptive trapping of the virus [37]. In addition, bacterial *lipopolysaccharides* (LPS), such as *Pseudomonas aeruginosa*, and *Salmonella enterica*, were found to bind and alter the morphology of AIV and disrupt the persistence and stability of AIV [40]. On the other hand, the gut microbiome can indirectly suppress AIV infection by initiating IFN-I responses to drive host antiviral immunity, inducing apoptosis of infected cells, and promoting the elimination of AIV [38,44]. A study showed that IFN-α inhibited H9N2 AIV infection without affecting the gut microbiome [45]. The microbiome could modulate the immune deficiency of CD4/CD8 T cells due to AIV infection and induce B cells to produce IgA to neutralize viral infectivity [40]. Meanwhile, probiotics in the intestine can also stimulate the activity of antigen-presenting cells (APCs) and natural killer (NK) cells, creating a barrier in the intestinal epithelium, and improving the defense against AIV infection [41]. The interactions between AIV infection and the gut microbiome of poultry are shown in Fig. 2.

**Table 2:** **Possible mechanisms of suppression of AIV infection by the poultry gut microbiome**.

	Possible mechanisms	References
Direct suppression	Suppressing AIV replication	[35,36]
	Preventing AIV from entering host cells	[37]
	Disrupting the persistence and stability of AIV	[18]
Indirect suppression	Initiating IFN-I response and driving systemic antiviral immunity	[38,39]
	Modulating the immune responses of CD4/CD8 T cells and B cells	[40]
	Stimulating antigen-presenting cells and enhancing NK cell activity	[41]

*Note:* IFN-I represents type I interferon; NK cell represents natural killer cell.

Moreover, poor farm and market environments could create new opportunities for AIV mutational recombination, which would become “hotbeds” for the spread of AIV [46], [47], [48]. A study monitoring AIV infection in a live poultry market in Bangladesh found that environmental samples from the market were positive for AIV throughout the year, and AIV (including subtypes H5N1 and H9N2) was also found in the backyard and small-scale reared poultry [46]. High ambient temperature (36 °C) exposure also would suppress the host's adaptive immune response to influenza A virus infection [47]. A study of the geographic distribution of AIV in poultry in China from 2014 to 2016 also found that AIV prevalence showed an increasing trend from north to south [49]. Meanwhile, wet agricultural systems in southern regions could further promote the interaction between poultry and wild birds, increasing the prevalence of avian influenza [50]. It is suggested that the rearing environment, climatic characteristics, and type of agricultural system may indirectly influence the disease risk of avian influenza during transmission, as the distribution of the AIV is influenced by local climate change and population size.

### 2.2 Human-environment interface

**Fig. 3:**
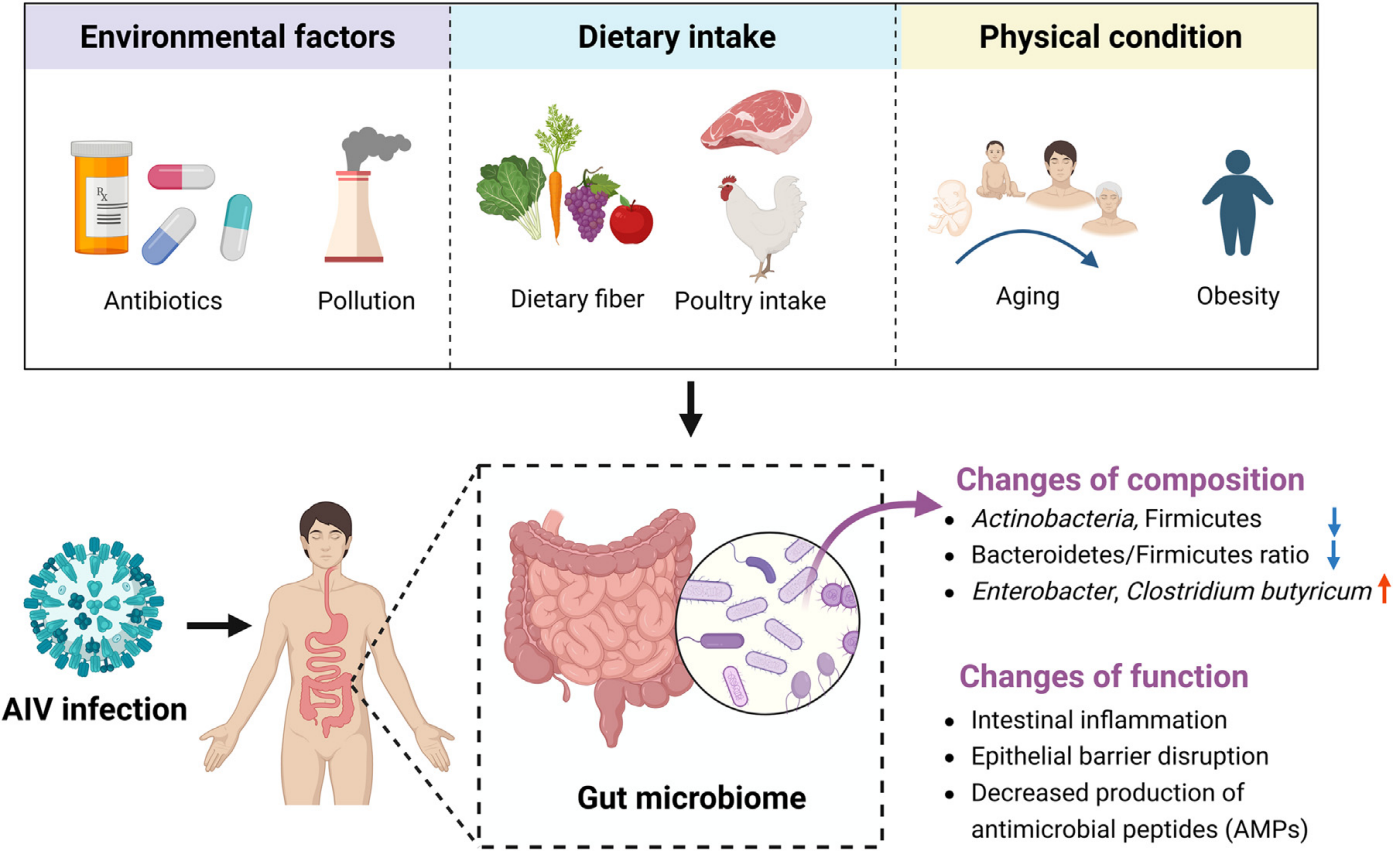
**Changes of composition and function in the human gut microbiome during AIV infection.** Note: Antibiotics, pollution, dietary fiber, poultry intake, aging, and obesity could affect the human gut microbiome. When humans are infected with AIV, the composition and function of the gut microbiome change.

Among the factors involved in the alteration of the human gut microbiome composition and functionality are environmental factors (e.g., antibiotics, pollution), dietary intake (e.g., dietary fiber, poultry intake), and physical condition (e.g., aging, obesity) [51,52]. For the human gut microbiological environment, the human gut contains up to 100 trillion (10^14^) microorganisms, which change dynamically as people get older [51]. The adult gut microbiome is more mature and stable, and is mainly dominated by *Bacteroidetes, Methanobrevibacter smithii*, and *Firmicutes*, of which 95% of *Firmicutes* sequences belong to *Clostridia* class [51,53]. Previous studies have shown that the human gut microbiome was strongly associated with AIV infections [54,55]. A study found that the relative abundance of intestinal *Actinobacteria* and *Firmicutes* was significantly reduced in H1N1 patients at the phylum level compared with healthy participants [54]. Infections by the H1N1 subtype of AIV led to a decrease in the Bacteroidetes/Firmicutes ratio [19]. In another study, an imbalance between *Bifidobacterium* and *Enterobacteriaceae* was observed in patients who were infected with H7N9 avian influenza, with the level of *Bifidobacterium* usually being higher or equal to *Enterobacteriaceae* [55]. Moreover, in patients infected with H7N9, the diversity of the intestinal microbiome was reduced, while the enrichment in some bacteria was obtained such as *Enterobacter*, and *Clostridium butyricum* regardless of the use of antibiotics [56]. Significantly, *Enterobacter* plays an important role in diseases of the gastrointestinal tract, lower respiratory tract, and lung infections [57]. AIV infection alters the function of the gut microbiome, including intestinal inflammation, epithelial barrier disruption, and reduced production of antimicrobial peptides (AMPs), leading to ecological dysbiosis of the intestinal microbiome [58]. We summarized the changes in the human gut microbiome during AIV infection in Fig. 3.

Under the background of climate change, the aerosol transmission pathway further aggravates the spread of influenza [59]. Studies have confirmed that air pollution is associated with an increased risk of influenza outbreak and transmission [60]. Furthermore, dust and particulate matter can serve as vectors allowing AIV to be transported for a long distance, which could also increase the risk of avian-origin human influenza infection, suggesting climate change may have unforeseen and dramatic effects on human health [61].

### 2.3 Poultry-human interface

A large number of infectious diseases including avian influenza, are zoonotic, which means that most of them originate in animals and can be transmitted to humans. Avian influenza is a classic case of environmental factors driving the thriving of humans and animals. Wild birds, especially waterfowl, are natural reservoirs for avian influenza and often interact with poultry during migration for breeding and foraging. While wild birds are typically asymptomatic carriers of low pathogenic AIV, when poultry is infected with AIV, it can trigger outbreaks of avian influenza on all continents [62].

AIV infection directly in humans is relatively unlikely due to receptor specificity. In poultry, the main site of AIV replication is the gastrointestinal tract, whereas in humans, infectious particles must reach the respiratory tract to trigger infection [63]. Furthermore, the optimal temperature for virus replication constitutes an additional barrier to zoonotic transmission of AIV, as AIV tends to replicate at higher temperatures (greater than 37 °C), but the temperatures in the human upper respiratory tract are relatively lower (∼33 °C) [64]. However, most evidence of AIV infection in humans is associated with the H5 and H7 subtypes, suggesting that mutations and recombination may enable the transmission of viruses of different lineages in humans [65]. Furthermore, intermediate hosts, such as pigs, may help AIV adapt to ‘human-type' receptor specificity and have a higher binding affinity, thereby facilitating AIV infection of humans across the species barrier [66,67].

## 3 Monitoring avian influenza with the “One Health” approach

Many experts and organizations have recognized that AIV might be the progenitors of the next human pandemic virus and surveillance is essential for preventing and controlling potential outbreaks [68]. This requires full support from the international and scientific community to track and investigate AIV in detail and promptly. As a key component of the entire Earth's ecosystem, the microbiome participates in various biogeochemical cycles, such as carbon, nitrogen, and sulfur cycles. It also affects the health of humans and other living organisms through various mechanisms, such as regulating immune responses, producing metabolites, and competing with pathogens. Therefore, effective monitoring of the gut microbiome needs to be strengthened to safeguard the common health of the environment, animals, and humans.

### 3.1 International practices in avian influenza surveillance

#### 3.1.1 Global practices

Global Influenza Surveillance and Response System (GISRS) has been proven valuable, as it is a system to protect people from the threat of influenza [69]. Up to June 2023, GISRS consists of more than 150 institutions from 127 World Health Organization (WHO) Member States, including National Influenza Centers (NICs), WHO Collaborating Centers, and Essential Regulatory Laboratories. This system monitors, prepares, and responds to global zoonotic influenza, and alerts for novel AIV. Recognizing the interrelatedness and complexity of zoonotic diseases, such as avian influenza, the Global Response to avian influenza initiated an unprecedented collaboration among the WHO/the Food and Agriculture Organization (FAO)/World Organization for Animal Health (OIE), which advocated for a holistic and multidisciplinary approach to address the health risk at the Poultry-Environment-Human Interface, such as foodborne, neglected zoonotic and tropical diseases [70].

#### 3.1.2 China

The H5N1 avian influenza outbreak that occurred in Hong Kong, China in 1997, the Government played a key role in the prevention and control of the outbreak by implementing a series of measures. A series of measures were taken to improve the hygiene and biosecurity of farms and live poultry markets, such as collecting samples, conducting surveillance, and monitoring for influenza viruses [71]. Over the same period, mainland China faced more challenges from H7N9 avian influenza, which had higher mortality, wider distribution, and longer duration than H5N1. However, the number of H7N9 infections has decreased significantly in mainland China since 2018, partly due to the government's efforts to adopt the “One Health” strategy. The government has established a comprehensive framework for joint avian influenza prevention and control, involving multiple sectors such as health, agriculture, environment, and wildlife. Some of the key measures include managing and monitoring AIV in poultry and wild birds, vaccinating poultry against AIV, and raising public awareness and education about avian influenza. Since 2013, rare new subtypes of animal influenza virus infections have been identified timely through the Chinese surveillance systems for the influenza pandemic, including the first H10N8 avian influenza infections that were detected in Jiangxi Province in 2013 [72].

#### 3.1.3 Other countries

To prevent and respond to avian influenza in poultry and wild birds, the United States established the National Animal Health Laboratory Network (NAHLN) in 2002 and released the National Avian Influenza Surveillance Program (NAISP) in 2007. The power of the NAHLN demonstrated its capability to test large numbers of AIV samples and send standardized data streams automatically to a national repository during 2014–2015, which resulted in the loss of about 50 million chickens and turkeys [73]. The NAISP is a collaborative and robust program involving United States policymakers, industry practitioners, and state stakeholders, which aims to achieve a high probability of detection or sensitivity for different subtypes of AIV. In addition, a United States Interagency Strategic Plan was developed and implemented to monitor the risk of AIV introduction via wild birds [74].

### 3.2 Major challenges in the current avian influenza surveillance system

#### 3.2.1 Absence of a global collaborative network platform for microbiome-related data

Multisectoral cooperation among countries is in place for some of the AIV outbreaks that have occurred or are occurring, but few involved microbiome-associated and wildlife-associated avian influenza in routine field testing. Global platforms for sharing surveillance data openly and transparently are even scarce. Meanwhile, many surveillance stations lack professional staff and inadequate infrastructure to detect pathogens and microbiomes quickly and accurately.

#### 3.2.2 Insufficient scientific research and technology on microbiome-related avian influenza

The active surveillance of microbiome-related avian influenza faces many challenges, such as limited specialized resources, the lack of a globally standardized testing process, weak scientific research strength of national institutions, poor data sharing and utilization. These factors hinder the analysis of large numbers of microbiome samples and the translation of scientific findings into policy actions.

#### 3.2.3 Lack of monitoring and response mechanisms for areas at high risk of spillover of new avian influenza outbreaks

Climate change and land-use change were reported to increase the frequency and intensity of wildlife-poultry-human interactions in some areas, which in turn promoted the risk of spillover and outbreaks of zoonotic diseases, especially avian influenza [75]. However, there lacks of a systematic mechanism to assess regional risks and enhance related monitoring and response, which hampers the preparedness for a possible pandemic of avian influenza in human societies.

### 3.3 Suggestions to improve the microbiome-related avian influenza surveil-lance system based on the “One Health” concept

**Fig. 4:**
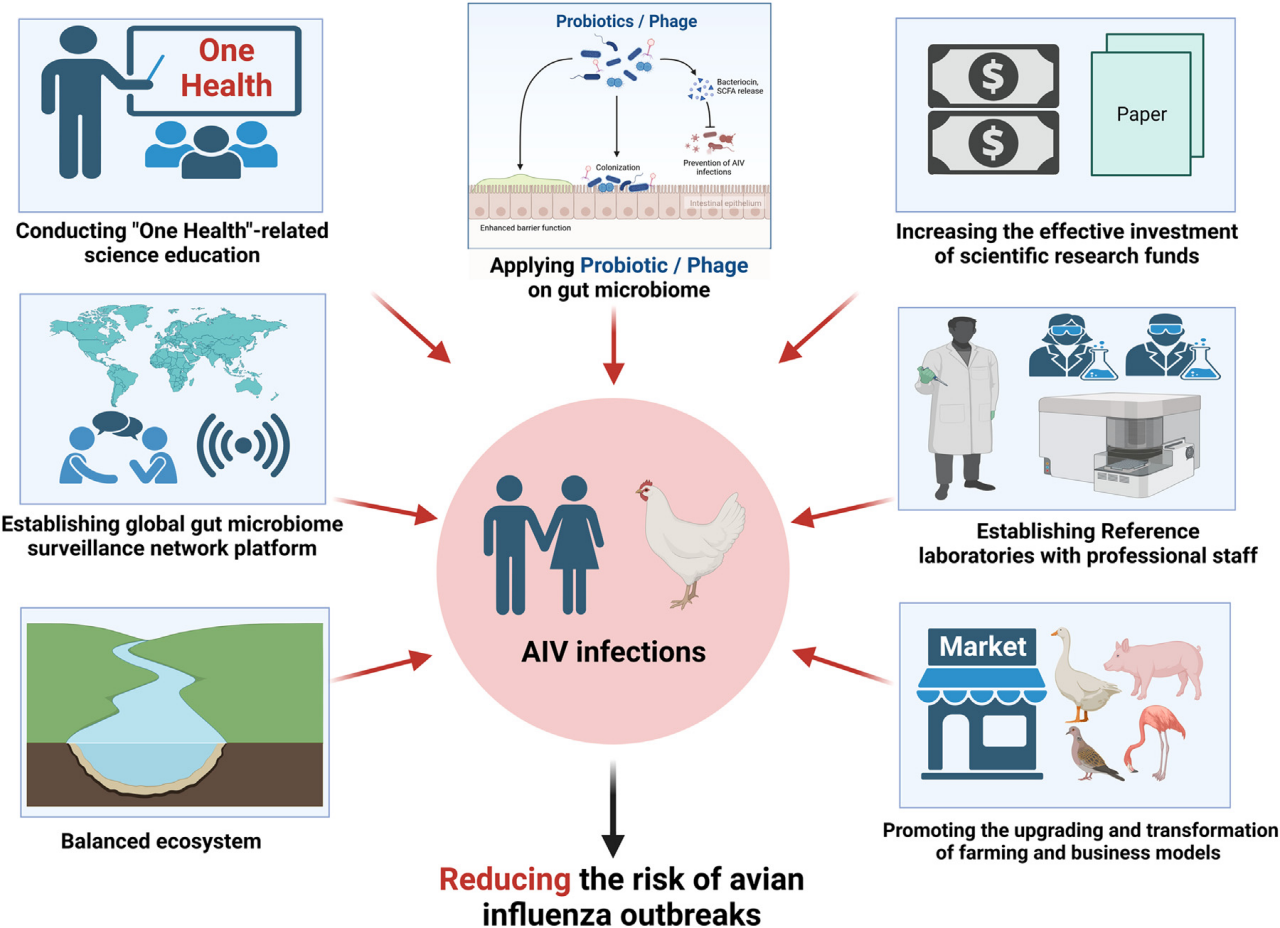
**Suggested strategies for reducing the risk of avian influenza outbreaks based on the “One Health” concept**.

Ending the pandemic does not mean that avian influenza will never happen again. Instead, a worldwide pandemic should be contained at a faster speed and a smaller cost in the future. Concerning an unpredictable pandemic like COVID-19, it is time to take strategies to improve avian influenza surveillance systems based on the “One Health” concept. The possible suggestions for reducing the risk of avian influenza outbreaks can be found in Fig. 4.

#### 3.3.1 Applying microbiological reagents

Phage agents and probiotics can help maintain the gut microbial balance, which may be the irreplaceable weapon to block the chain of transmission of avian influenza in the first place. They can achieve this by targeting the dominant pathogenic strains, such as *Salmonella* and *Campylobacter*, and preserving the diversity and functionality of the microbial community [76,77]. Phage agents work by selectively killing abundant and harmful bacteria, while probiotics and prebiotics work by enhancing the immunity and feeding efficiency of poultry and humans [78], [79], [80]. These strategies can improve gut microbiome homeostasis and lower the risk of invasive infections.

#### 3.3.2 Establishing a global gut microbiome surveillance network platform

Establishing a global collaborative network platform can improve the surveillance and response to avian influenza. This platform can collect and analyze microbiome samples from different populations of humans and animals across regions and seasons. It can also enable rapid identification of AIV mutations and gut microbiome changes, and support clinical trials of vaccines and drugs [68]. This can help us understand the diversity and dynamics of the gut microbiome, identify potential biomarkers and indicators of health and disease, and develop novel strategies and interventions to modulate the gut microbiome for better health outcomes. Some possible actions include establishing sentinel sites for poultry surveillance and proactive cohorts of animal workers, and creating reference laboratories with standardized testing processes and functions.

#### 3.3.3 Promoting the upgrading and transformation of farming and business models

To prevent and control avian influenza, the poultry industry should reform its supply chain and retail model, and adopt more intensive, efficient, standardized, and ecological farming practices. Biosecurity, veterinary, and reporting systems should be improved in poultry farms. Live poultry trading and transport should be regulated to reduce human-animal contact. Wildlife-poultry contact should also be avoided.

#### 3.3.4 Conducting “One Health”-related science education

Scientific education is essential to promote the “One Health” concept. The public should learn the basics of avian influenza, the interconnection of human, animal, and environmental health, and the personal responsibility for protecting their own and others’ health. Moreover, the occupational populations, especially farmers, should improve their health literacy and reduce their exposure and infection risk to AIV.

## 4 Conclusion

This review discusses the interactions and mechanisms of AIV infection and gut microbiome in poultry and humans from a “One Health” perspective. We highlight that the gut microbiome and AIV have a close and mutual influence on the health of poultry, humans, and the environment. However, various factors, such as climate change, urbanization, ecosystem imbalances, and globalization, can increase the risk and frequency of avian influenza outbreaks, and the composition and function of poultry and human gut microbiome are more susceptible to change. Therefore, we recommend strengthening the monitoring of the gut microbiome to protect the health of the environment, animals, and humans under the “One Health” concept.

## Author contributions

**Ling Zhang:** Writing – original draft, Methodology, Visualization, Writing – review & editing. **Erkai Zhou:** Supervision, Formal analysis, Software. **Ce Liu:** Visualization. **Xiaoyu Tian:** Visualization. BaodeXue: Supervision. **Kai Zhang:** Investigation, Methodology, Supervision,Writing – review & editing. **Bin Luo:** Conceptualization, Writing – review & editing.

## Declaration of competing interest

The authors declare that they have no conflicts of interest in this work.
